# Effects of PCSK-9 Inhibition by Alirocumab Treatments on Biliary Cirrhotic Rats

**DOI:** 10.3390/ijms23137378

**Published:** 2022-07-02

**Authors:** Hui-Chun Huang, Shao-Jung Hsu, Ching-Chih Chang, Chiao-Lin Chuang, Ming-Chih Hou, Fa-Yauh Lee

**Affiliations:** 1 Faculty of Medicine, National Yang Ming Chiao Tung University, Taipei 112, Taiwan; hchuang2@vghtpe.gov.tw (H.-C.H.); sjhsu@vghtpe.gov.tw (S.-J.H.); clchuang@vghtpe.gov.tw (C.-L.C.); mchou@vghtpe.gov.tw (M.-C.H.); fylee@vghtpe.gov.tw (F.-Y.L.); 2 Division of Gastroenterology and Hepatology, Department of Medicine, Taipei Veterans General Hospital, Taipei 112, Taiwan; 3 Division of General Medicine, Department of Medicine, Taipei Veterans General Hospital, Taipei 112, Taiwan

**Keywords:** hepatic encephalopathy, liver cirrhosis, oxidized low-density lipoprotein, PCSK9 inhibitor

## Abstract

Hyperlipidemia and oxidative stress with elevated oxidized low-density lipoprotein (ox-LDL) exacerbate hepatic inflammation and fibrosis. The plasma level of low-density lipoprotein (LDL) is controlled by proprotein convertase subtilisin/kexin 9 (PCSK9). Alirocumab is a monoclonal antibody that decreases LDL via inhibiting PCSK9 function. Apart from lipid-lowering effects, alirocumab exerts anti-inflammation, anti-angiogenesis and anti-oxidant effects. This study aims to investigate the impact of alirocumab treatment on common bile duct ligation (BDL)-induced biliary cirrhotic rats. After a 4-week treatment of alirocumab, the hemodynamic data, blood biochemistry, ox-LDL level, oxidative stress markers, severity of hepatic encephalopathy and abnormal angiogenesis of BDL rats were measured and compared to the control group. BDL rats presented cirrhotic pictures and elevated ammonia, total cholesterol, LDL and ox-LDL levels compared to the control group. Alirocumab decreased plasma levels of total cholesterol, LDL, and oxidative stress markers; however, it did not affect the hemodynamics, liver and renal biochemistry, and the plasma levels of ammonia and ox-LDL. The motor activities, portal-systemic collaterals and mesenteric vascular density were not significantly different between alirocumab-treated and control groups. In addition, it did not affect hepatic inflammation, intrahepatic angiogenesis, liver fibrosis and free cholesterol accumulation in the liver of BDL rats. In conclusion, PCSK9 inhibition by alirocumab treatment ameliorates hyperlipidemia and systemic oxidative stress in biliary cirrhotic rats. However, it does not affect the plasma level of ox-LDL, intrahepatic inflammation and fibrosis. In addition, PCSK9 inhibition has a neutral effect on abnormal angiogenesis and hepatic encephalopathy in biliary cirrhotic rats.

## 1. Introduction

In liver cirrhosis, chronic inflammation and fibrosis increase intrahepatic resistance and then induce portal hypertension and stimulate the formation of collateral vessels, which causes hepatic encephalopathy [1]. Hepatic encephalopathy is characterized by neuropsychiatric and motor disturbance in patients with fulminant hepatic failure or liver cirrhosis [2]. Two major contributing factors of hepatic encephalopathy are abundant portal-systemic collaterals and hepatic dysfunction. Emerging data reveal that abnormal lipid homeostasis may contribute to the pathogenesis of hepatic inflammation and liver fibrosis [3,4]. Studies of non-alcoholic steatohepatitis show that hypercholesterolemia is associated with free cholesterol accumulation in hepatic stellate cells that triggers liver fibrosis [5]. Meanwhile, intrahepatic cholesterol accumulation is attributed to hepatocyte apoptosis, macrophage recruitment and liver fibrosis [6]. In addition to non-alcoholic fatty liver disease, high cholesterol intake also exaggerates acetaminophen-induced liver injury via free cholesterol accumulation in liver sinusoidal endothelial cells, demonstrating a novel role of free cholesterol as a metabolic factor of various liver injuries [7].

Plasma cholesterol circulates as a constituent of lipoprotein. Hypercholesterolemia is characterized by elevated levels of plasma low-density lipoprotein (LDL). LDL particles are removed from circulation mainly by hepatic uptake through the LDL receptors, which is followed by the endocytosis of the complex by endosomes [8]. Therefore, LDL receptor levels are crucial for maintaining the optimal cellular cholesterol balance. The liver contains about 70% of total LDL receptors in the body, making it the major organ responsible for the turnover of LDL in the circulation [9]. LDL receptor levels are controlled by convertase subtilisin/kexin 9 (PCSK9), which is an enzyme that binds to the LDL receptor and promotes its degradation in the lysosomal pathway [10]. The inhibition of PCSK9 prevents LDL receptors degradation, resulting in the increased availability of LDL receptors to facilitate LDL clearance from the blood. PCSK9 is not only involved in the degradation of the LDL receptors but has been shown to influence inflammatory and oxidative processes [11]. Chronic inflammation can stimulate the expression of PCSK9, causing the degradation of LDL receptors, which increases plasma LDL levels. The increased mRNA level of PCSK9 is found in the liver of cholesterol-fed mice treated with lipopolysaccharide [12]. In addition, the circulating PCSK9 is associated with steatosis grade, inflammation and fibrosis stage in non-alcoholic steatohepatitis patients [13]. On the other hand, the oxidized LDL (ox-LDL) has been proved to be associated with portal inflammation and fibrosis [3]. Lysosomal lipid accumulation in blood-derived macrophages can trigger ox-LDL-dependent murine hepatic inflammation in a non-alcoholic steatohepatitis murine model [14]. In chronic hepatitis C patients, the plasma level of ox-LDL is significantly higher than that in the normal controls, and it is strongly correlated to viral load, grade of inflammation and cirrhotic status [15].

**Figure 1:**
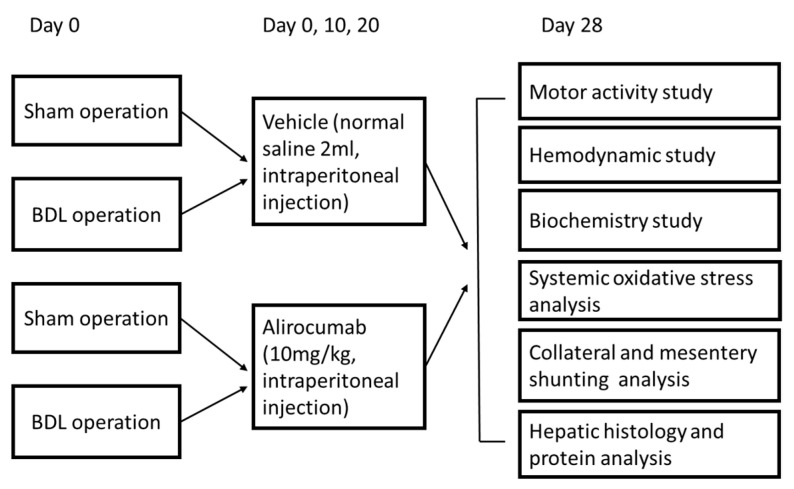
Study design of alirocumab-treated bile duct ligation (BDL) or sham-operated rats.

The impact of PCSK9 inhibition on liver cirrhosis remains unclear. Thus, we postulates that the inhibition of PCSK9 by alirocumab, a monoclonal antibody against PCSK9 [16], can decrease ox-LDL level, attenuate liver fibrosis, and intrahepatic inflammation; then, it consequently ameliorates cirrhosis-related complications. Thus, we design this study to test the therapeutic effect of alirocumab on cirrhotic rat. The study design is illustrated in Figure 1.

## 2. Results

### 2.1. Mortality Rates of Alirocumab- and Vehicle-Treated Rats

There was no significant difference in mortality rates between alirocumab- and vehicle-treated (control) common bile duct ligation (BDL) rats (alirocumab vs. control: 14.3% (2/14) vs. 8.3% (1/12), *p* > 0.05). All the sham-operated rats survived throughout the 4-week treatments.

### 2.2. Body Weight and Hemodynamic Parameters

Table 1 displays the body weight (BW) and hemodynamic parameters with or without alirocumab treatment. BDL rats had a significantly lower BW compared to sham rats (Sham + alirocumab (Sham-A, *n* = 12) vs. Sham + vehicle (Sham-V, *n* = 8) vs. BDL + alirocumab (BDL-A, *n* = 12) vs. BDL + vehicle (BDL-V, *n* = 11): 429 ± 26 vs. 420 ± 38 vs. 374 ± 23 vs. 384 ± 36 g; Sham-A vs. BDL-A and Sham-V vs. BDL-V: *p* < 0.05). In addition, BDL rats had significantly lower mean arterial pressure (MAP), higher portal pressure (PP), higher cardiac index (CI) and lower systemic vascular resistance (SVR) (MAP = 155 ± 9 vs. 145 ± 10 vs. 134 ± 14 vs. 132 ± 14 mmHg; PP = 8.6 ± 1.0 vs. 8.7 ± 0.8 vs. 16.4 ± 1.9 vs. 16.9 ± 2.6 mmHg; CI = 33.4 ± 5.0 vs. 28.7 ± 2.9 vs. 43.0 ± 6.4 vs. 44.1 ± 7.4 mL/min/100 g; SVR = 4.8 ± 0.7 vs. 5.1 ± 0.7 vs. 3.2 ± 0.5 vs. 3.1 ± 0.7 mmHg/mL/min/100 g; Sham-A vs. BDL-A and Sham-V vs. BDL-V: *p* < 0.05). The higher superior mesentery arterial flow (SMAf) and portal venous flow (PVf) with lower superior mesentery arterial resistance (SMAR) were also noted in BDL rats (SMAf = 6.2 ± 1.0 vs. 5.8 ± 0.6 vs. 8.4 ± 1.1 vs. 8.2 ± 1.6 mL/min/100 g; PVf = 8.9 ± 1.3 vs. 10.1 ± 0.9 vs. 12.9 ± 2.1 vs. 11.7 ± 1.7 vs. mL/min/100 g; SMAR = 24.2 ± 3.7 vs. 23.8 ± 2.8 vs. 14.3 ± 2.9 vs. 14.7 ± 4.1 mmHg/mL/min/100 g; Sham-A vs. BDL-A and Sham-V vs. BDL-V: *p* < 0.05). The aforementioned presentations in BDL rats were typical of hyperdynamic circulatory status in liver cirrhosis and portal hypertension. Alirocumab did not affect hemodynamic parameters in sham and BDL rats.

### 2.3. The Biochemistry Parameters and Ox-LDL

Table 2 displays the biochemistry parameter and ox-LDL in sham and BDL rats after alirocumab treatments (Sham-A vs. Sham-V vs. BDL-A vs. BDL-V: *n* = 12:8:12:11). Compared to sham-operated rats, BDL rats had higher total cholesterol, LDL, ox-LDL, ammonia, alanine aminotransferase (ALT), aspartate aminotransferase (AST) and total bilirubin levels (Sham-V vs. BDL-V: Total cholesterol = 52 ± 12 vs. 81 ± 9 mg/dL; LDL = 11 ± 2 vs. 45 ± 9 mg/dL; ox-LDL = 44 ± 10 vs. 714 ± 237 ng/mL; ammonia = 106 ± 26 vs. 235 ± 66 μmol/L; ALT = 55 ± 12 vs. 118 ± 37 IU/L; AST = 101 ± 51 vs. 567 ± 132 IU/L; Total bilirubin = 0.04 ± 0.03 vs. 8.4 ± 1.3 mg/dL; all *p* < 0.05). The plasma levels of total cholesterol and LDL were significantly decreased in sham-operated rats after alirocumab treatments (Sham-A vs. Sham-V: Total cholesterol = 35 ± 11 vs. 52 ± 12 mg/dL; LDL = 7 ± 4 vs. 11 ± 2 mg/dL; both *p* < 0.05). Similarly, alirocumab also reduced plasma levels of total cholesterol and LDL in BDL rats (BDL-A vs. BDL-V: Total cholesterol = 62 ± 14 vs. 81 ± 9 mg/dL; LDL = 31 ± 9 vs. 45 ± 9 mg/dL; both *p* < 0.05). Although alirocumab reduced total cholesterol and LDL in sham-operated and BDL rats, it did not alter ox-LDL, ammonia, ALT, AST and total bilirubin levels.

### 2.4. Motor Activity of BDL Rats with or without Alirocumab Treatment

Table 3 presents the motor activities of BDL rats with or without alirocumab treatment (BDL-V vs. BDL-A: *n* = 11:12). There were no significant differences in traveled distance, resting time, ambulatory time and stereotypic time between the alirocumab-treated and control BDL rats (all *p* > 0.05).

### 2.5. Histopathological Change, Intrahepatic Angiogenesis and Free Cholesterol Accumulation of Liver

**Figure 2:**
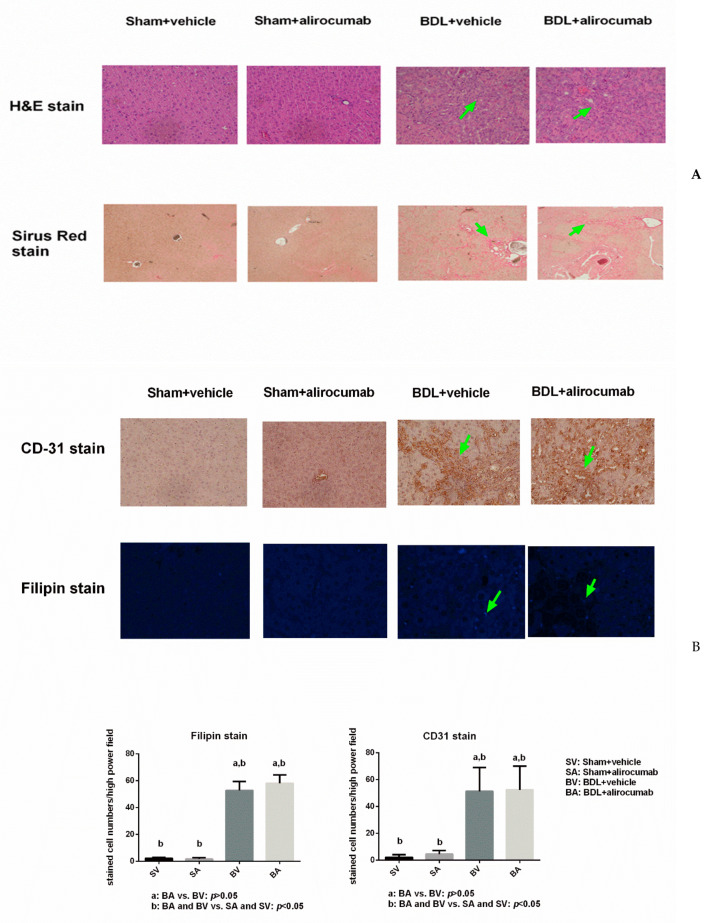
(**A**) Liver histology of sham or BDL rats with or without alirocumab treatment. The representative H&E staining image of sham rats showed normal architecture of liver tissue. In contrast, the liver of BDL rats presented many inflammatory cells and bile duct proliferation (green arrow, magnification 200×). Sirius Red staining revealed the obvious fibrosis of liver (green arrow) in BDL rats, which was not attenuated by alirocumab (magnification 40×). (**B**) The immunohistochemical staining of liver in sham and BDL rats with or without alirocumab treatments. There were many CD31-positive staining cells (green arrow indicating brown cells) and free cholesterol droplet (green arrow indicating light blue cells) in the liver of BDL rats (magnification 200×), indicating the increased intrahepatic angiogenesis and steatosis. In contrast, there are scanty CD31-positive staining cells and free cholesterol droplet in the liver of sham rats. Alirocumab did not reduce the numbers of CD31-staining cells and filipin-staining droplets in BDL and sham rats (Sham-A vs. Sham-V vs. BDL-A vs. BDL-V: *n* = 12:8:12:11; Sham-A and Sham-V vs. BDL-A and BDL-V, *p* < 0.05, BDL-A vs. BDL-V, *p* > 0.05).

Figure 2A depicts the histopathological changes of sham and BDL rats with or without alirocumab treatment. Compared to sham-operated rats, the hepatic H&E staining shows many mononuclear cells infiltration, hepatocytes necrosis and bile duct proliferation, indicating the inflammatory change in the liver of BDL rats, which was not ameliorated by alirocumab. Sirius Red staining revealed obvious fibrosis of liver in BDL rats, which was not attenuated by alirocumab, either. Figure 2B reveals many CD31-positive staining cells in the liver of BDL rats compared to the control group, indicating an increased intrahepatic angiogenesis of BDL rats. Alirocumab treatment did not affect the numbers of CD31-staining cells. Similarly, the liver of BDL rats showed more free cholesterol accumulations demonstrated by filipin staining compared to the sham-operated rats. However, alirocumab treatment did not attenuate the cholesterol accumulation.

### 2.6. Portal-Systemic Shunting Degree and Mesenteric Vascular Density in BDL Rats with or without Alirocumab Treatment

**Figure 3:**
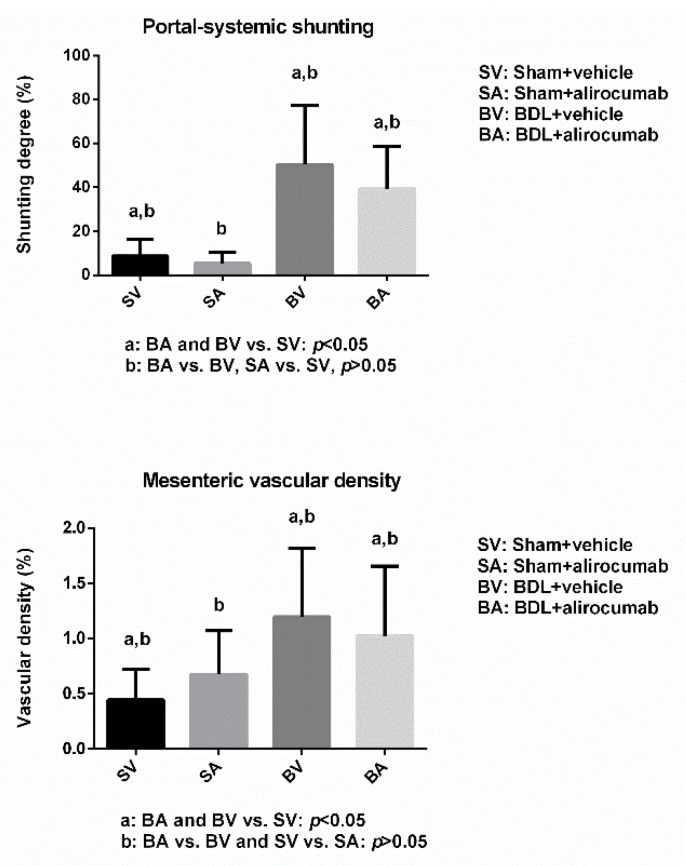
The portal-systemic collateral shunting and mesenteric vascular density of sham or BDL rats with or without alirocumab. The collateral shunting degree significantly increased in BDL rats compared to sham rats, which was not influenced by alirocumab, both in sham and BDL rats (Sham-V vs. Sham-A vs. BDL-V vs. BDL-A: *n* = 8:9:9:10; BDL-A and BDL-V vs. Sham-V, *p* < 0.05, BDL-A vs. BDL-V, *p* > 0.05, Sham-A vs. Sham-V, *p* > 0.05). Similarly, the mesenteric vascular density significantly increased in BDL rats, which was not influenced by alirocumab (BDL-A and BDL-V vs. Sham-V, *p* < 0.05, BDL-A vs. BDL-V, *p* > 0.05, Sham-A vs. Sham-V, *p* > 0.05).

Figure 3 shows the degree of portal-systemic shunting and mesenteric vascular density in sham or BDL rats with or without alirocumab (Sham-V vs. Sham-A vs. BDL-V vs. BDL-A: *n* = 8:9:9:10). The portal-systemic shunting degrees significantly increased in BDL rats compared to those in sham rats. However, alirocumab did not affect collateral shunts in sham and BDL rats (Sham-V vs. Sham-A vs. BDL-V vs. BDL-A: 8.7 ± 7.6 vs. 5.3 ± 5.2 vs. 50.4 ± 27.2 vs. 39.3 ± 19.2 %; BDL-V and BDL-A vs. Sham-V, *p* < 0.05; BDL-V vs. BDL-A and Sham-V vs. Sham-A, both *p* > 0.05). Similarly, BDL rats had higher mesenteric vascular densities compared to those in sham rats. Alirocumab treatment did not change mesenteric vascular density in both sham and BDL rats (Sham-V vs. Sham-A vs. BDL-V vs. BDL-A: 0.45 ± 0.28 vs. 0.68 ± 0.39 vs. 1.20 ± 0.62 vs. 1.12 ± 0.64 %; BDL-V and BDL-A vs. Sham-V, *p* < 0.05; BDL-V vs. BDL-A and Sham-V vs. Sham-A, both *p* > 0.05).

### 2.7. Effects of Alirocumab Treatment on Systemic Oxidative Stress

**Figure 4:**
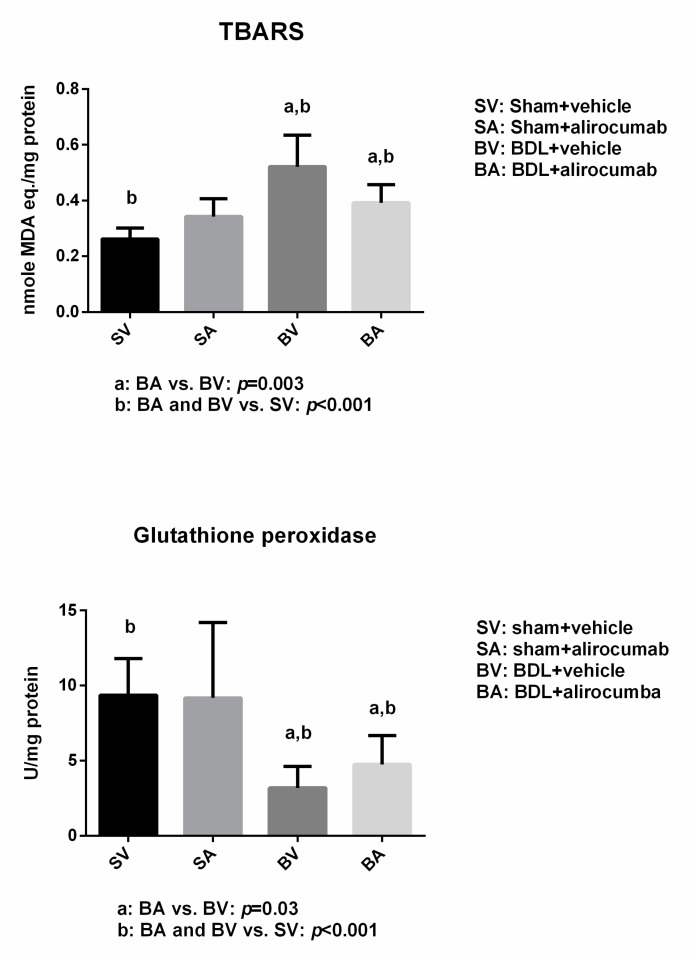
Oxidative stress in sham and BDL rats with or without alirocumab treatment. Up-regulation of the oxidative stress marker, thiobarbituric acid reactive substances (TBARS) activity (Sham-V vs. Sham-A vs. BDL-V vs. BDL-A: *n* = 8:12:11:12; BDL-A vs. BDL-V, *p* = 0.003, BDL-A and BDL-V vs. Sham-V, *p* < 0.001), and down-regulation of anti-oxidative stress marker, glutathione peroxidase activity (BDL-A vs. BDL-V, *p* = 0.03, BDL-A and BDL-V vs. Sham-V, *p* < 0.001) were noted in BDL rats compared to sham rats. Alirocumab significantly reduced TBARS and elevated glutathione peroxidase activities in BDL rats.

Figure 4 depicts the systemic oxidative stress markers of sham and BDL rats treated by alirocumab (Sham-V vs. Sham-A vs. BDL-V vs. BDL-A: *n* = 8:12:11:12). Up-regulated thiobarbituric acid reactive substances (TBARS) and down-regulated glutathione peroxidase were found in BDL rats compared to those in sham rats. Furthermore, alirocumab significantly down-regulated the TBARS and up-regulated glutathione peroxidase in BDL rats (Sham-V vs. Sham-A vs. BDL-V vs. BDL-A: TBARS = 0.26 ± 0.04 vs. 0.34 ± 0.06 vs. 0.52 ± 0.11 vs. 0.39 ± 0.07 nmole MDA eq./mg protein; Sham-V, Sham-A vs. BDL-V, BDL-A, *p* < 0.001; BDL-V vs. BDL-A, *p* = 0.003; glutathione peroxidase = 9.4 ± 2.4 vs. 9.2 ± 5.0 vs. 3.2 ± 1.4 vs. 4.8 ± 1.9 U/mg protein; Sham-V, Sham-A vs. BDL-V, BDL-A, *p* < 0.001; BV vs. BA, *p* = 0.03). TBARS is a marker of oxidative stress, and glutathione peroxidase is an anti-oxidative marker. Since alirocumab treatment significantly down-regulated the TBARS and up-regulated glutathione peroxidase in BDL rats, it indicates the attenuation of oxidative stress by alirocumab.

### 2.8. Hepatic Protein Expressions in the Liver of BDL Rats

**Figure 5:**
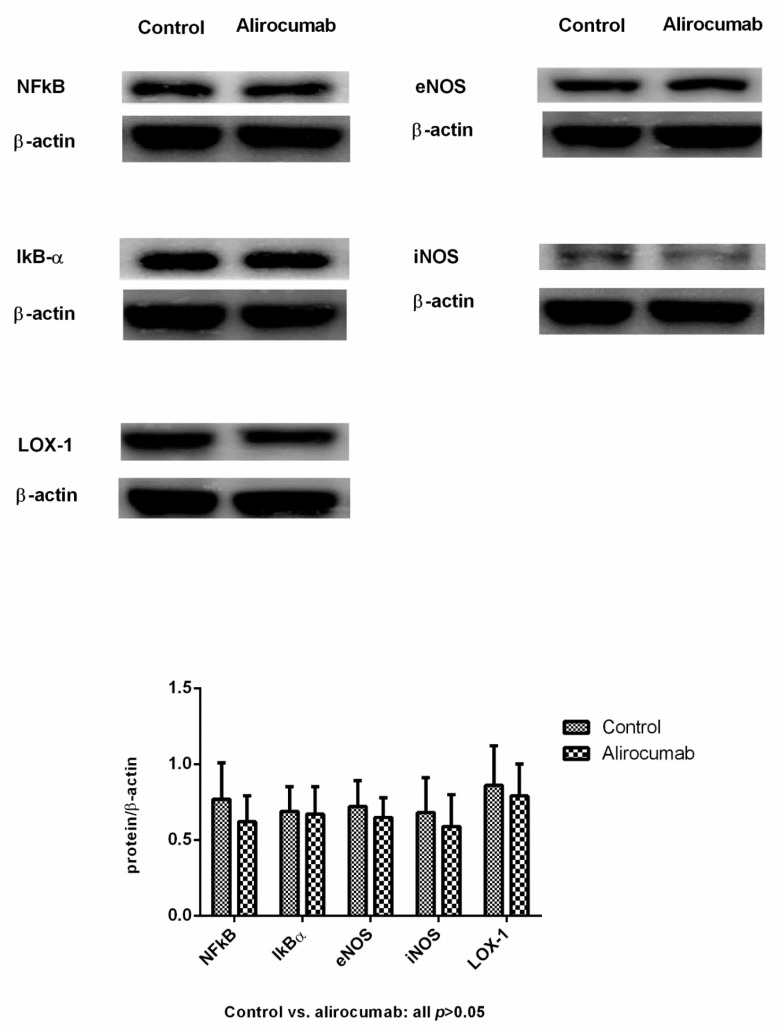
Hepatic protein expressions of BDL rats with or without alirocumab treatment. The NFkB, IkBα, eNOS, iNOS, and LOX-1 protein expressions were not significantly influenced by alirocumab (BDL-V vs. BDL-A: *n* = 11:12, BDL-V vs. BDL-A, all proteins *p* > 0.05).

Figure 5 reveals the hepatic protein expressions of BDL rats (BDL-V vs. BDL-A: *n* = 11:12). The NFκB, IκB, eNOS, iNOS and LOX-1 protein expressions were not significantly influenced by alirocumab (NFκB/β-actin = 0.77 ± 0.24 vs. 0.62 ± 0.17; IκB/β-actin = 0.69 ± 0.16 vs. 0.67 ± 0.18; eNOS/β-actin = 0.72 ± 0.17 vs. 0.65 ± 0.13; iNOS/β-actin = 0.68 ± 0.23 vs. 0.59 ± 0.21; LOX-1/β-actin = 0.86 ± 0.26 vs. 0.79 ± 0.21; all *p* > 0.05).

## 3. Discussion

In the present study, we highlight for the first time that the inhibition of PCSK9 by a monoclonal antibody, alirocumab, can attenuate systemic oxidative stress of cirrhotic rats. In accordance to our previous findings, cirrhotic rats had significant jaundice, portal hypertension, hyperdynamic circulation and increased portal-systemic shunts compared to sham-operated rats [17]. Meanwhile, emerging evidence demonstrated that chronic hepatic inflammation induced hyperlipidemia and enhanced oxidative stress [18]. Our data showed that alirocumab treatment decreased the plasma levels of total cholesterol and LDL cholesterol accompanied with attenuation of oxidative stress in cirrhotic rats. However, it did not reduce the plasma level of ox-LDL. In addition, alirocumab neither improved portal hypertension nor decreased portal-systemic collateral shunting in biliary cirrhotic rats.

The impact of hyperlipidemia on liver cirrhosis remains debated. Although hypercholesterolemia plays a crucial role in hepatic inflammation and fibrosis, the plasma cholesterol and lipoproteins levels tend to decrease with chronic liver disease [19]. The impairment of lipid metabolism is a common finding in cirrhotic patients [20]. In a retrospective cohort study of veterans with a new diagnosis of cirrhosis, hypercholesterolemia is associated with well-preserved hepatic function, and every 10 mg/dL increase in baseline total cholesterol is associated with a 3.6% decrease in mortality [21]. Some researchers suggest that the plasma level of cholesterol in patients with cirrhosis is inversely correlated with the severity of liver cirrhosis [22]. However, a contrary report shows that the LDL level is higher in patients with severe non-alcoholic liver cirrhosis [23]. Whether the lower plasma level of cholesterol is the aggravating factor or the consequence of hepatic failure in cirrhotic patients remains unclear. Our data showed that PCSK9 inhibition by alirocumab ameliorated hyperlipidemia, but it did not improve the hepatic inflammation and liver fibrosis. It is worth noting that intrahepatic free cholesterol accumulations was not altered by alirocumab in BDL rats, indicating that the improvement of hyperlipidemia by alirocumab was not accompanied by amelioration of intrahepatic steatosis.

Alirocumab significantly attenuated the systemic oxidative stress in BDL-induced cirrhotic rats. A recent report showed that lipid-lowering agents had anti-oxidant effect related to PCSK-9 inhibition [24]. However, the plasma level of ox-LDL level could not be reduced by alirocumab in the present study. The oxidative stress in prolonged cholestasis may enhance the oxidative modification of LDL within the liver of BDL rats [25]. Using BDL-induced biliary cirrhotic murine model, Yu et al. showed that rosuvastatin regulated the expression of intrahepatic ox-LDL and improved liver fibrosis [26]. Comert et al. also showed that ox-LDL accumulated in the liver of BDL mice [27]. The ox-LDL can induce tissue factor expression in T-lymphocytes via activation of lectin-like oxidized low-density lipoprotein rececptor-1 (LOX-1), and the attenuated LOX-1 expression also indicates the decrease in ox-LDL [28]. In addition, the modulatory role of LOX-1 on nitric oxide and the renin–angiotensin–aldosterone system as well as on fibrosis, apoptosis and inflammatory pathways has also been documented [29]. In the present study, alirocumab did not reduce the plasma level of ox-LDL and the expressions of intrahepatic LOX-1, iNOS and eNOS proteins, indicating its neutral effects on ox-LDL modulation. The cause that alirocumab ameliorates systemic oxidative stress without significantly influencing ox-LDL remains unknown. The possible explanation might be ascribed to the condition that the rapid and progressive cirrhotic change in 4 weeks after BDL operation may be too overwhelming to be ameliorated by merely PCSK9 inhibition. Further investigation, indeed, is required.

Although many reports show that alirocumab exerts anti-inflammation and anti-fibrosis capacities [3,11,12,13], the liver biochemistry and histopathology were not significantly different between alirocumab-treated and control BDL rats in the present study. The intrahepatic inflammatory indicators, such as NFκB and IκB proteins expressions, were not affected by alirocumab treatment as well. The hepatic fibrosis was not ameliorated, either. Overall, our data suggested the neutral effect on hepatic inflammation and intrahepatic fibrosis by alirocumab treatment. The current study design is a preventive rather than therapeutic approach. According to our data, alirocumab did not show anti-inflammation and anti-fibrosis effects on biliary cirrhosis. In contrast to our finding, Lee et al. have reported that alirocumab treatment improves alcohol-induced steatohepatitis in a murine model through the regulation of lipid metabolism, hepatic inflammation, and neutrophil infiltration [30]. Indeed, it is interesting to investigate the effect of alirocumab on liver damage derived from different mechanisms in the future. In addition, it is worth noting that the hepatotoxicity of alirocumab raises some concerns, since a retrospective study reveals that 6-months alirocumab treatment impairs liver function in patients with hyperlipidemia [31]. However, a contrary report shows that alirocumab treatment is safe in uremic patients under hemodialysis [32]. Therefore, more studies about the safety profile of alirocumab and its effect on different etiologies of liver injury are warranted.

BDL rats had abundant portal-systemic collaterals and mesenteric vasculatures. Our data showed that alirocumab did not diminish extrahepatic portal-systemic collaterals, mesentery vascular densities and intrahepatic angiogenesis in BDL rats. Since these crucial factors attributed for the pathogenesis of hepatic encephalopathy were not ameliorated, it was not surprising that alirocumab did not correct hepatic encephalopathy in BDL rats. A recent report showed that PCSK9 inhibition could inhibit tumor progression and improve survival in mice bearing colon cancer through anti-angiogenesis and anti-inflammation [33]. However, another report showed that the total obliteration of PCSK9 impaired liver regeneration after partial hepatectomy of mice, indicating that upon hepatic damage, lacking PCSK9 could be risky [34]. Therefore, more studies are warranted to clarify this issue regarding angiogenesis and PCSK9 inhibition.

In conclusion, PCSK9 inhibition by alirocumab ameliorates systemic oxidative stress and hyperlipidemia in BDL-induced cirrhotic rats. However, it did not improve the portal hypertension, hepatic inflammation, fibrosis, angiogenesis, intrahepatic steatosis and hepatic encephalopathy. The safety profile of alirocumab seems acceptable in cirrhotic status according to our data. However, it is necessary to conduct clinical trials to test the long-term effect of alirocumab treatment on cirrhotic patients in the future.

## 4. Materials and Methods

### 4.1. Animal Model for Biliary Cirrhosis

Male Sprague–Dawley rats weighing 240–280 g at the time of surgery were used for experiments. The rats were housed in a plastic cage and allowed free access to food and water. All rats were fasted for 12 h before the operation. Rats with secondary biliary cirrhosis were induced by common bile duct ligation (BDL) [35]. A high yield of secondary biliary cirrhosis was noted 4 weeks after the ligation [36]. The control group received sham operation without ligation of common bile duct. This study was authorized by the Animal Committee of our hospital (IACUC 2019-194). All animals received humane care according to the criteria outlined in the “Guide for the Care and Use of Laboratory Animals, 8th edition, 2011” published by the National Research Council, the United States.

### 4.2. Study Protocol

One day before BDL or sham operation, the rats were randomly divided into four groups: (1) Sham-operated rats administered with normal saline (vehicle); (2) Sham-operated rats administered with alirocumab; (3) BDL rats administered with normal saline and (4) BDL rats administered with alirocumab (10 mg/kg, intraperitoneal injection) on day 0, day 10 and day 20 post operation [37]. The survival rates were monitored. On the 28th day, the motor activities and hemodynamic data were measured. The blood was drawn for measuring oxidative stress, total cholesterol, LDL, ox-LDL, alanine aminotransferase (ALT), aspartate aminotransferase (AST), total bilirubin and creatinine levels at the end of experiments. The histopathology and hepatic protein expressions were investigated. In addition, on the parallel 4 groups, the effects of alirocumab treatment on the severity of portal-systemic collaterals and the severity of mesenteric angiogenesis were measured.

### 4.3. Measurement of Systemic and Portal Hemodynamics

The right femoral artery and superior mesentery vein were cannulated with PE-50 catheters that were connected to a Spectramed DTX transducer (Spectramed Inc., Oxnard, CA, USA). Continuous recordings of mean arterial pressure (MAP), heart rate (HR) and portal pressure (PP) were performed on a multi-channel recorder (model RS 3400, Gould Inc., Cupertino, CA, USA). Cardiac output (CO, mL/min) was measured by a thermodilution method as previously described [38]. Cardiac index (CI, mL/min/100 g body weight) was calculated as CO per 100 g body weight (BW). Systemic vascular resistance (SVR, mmHg/mL/min/100 g BW) was calculated via MAP divided by CI. Superior mesentery arterial resistance (SMAR, mmHg/mL/min/100 g BW) was calculated by (MAP-PP)/superior mesentery artery flow (SMAf) per 100 g BW. The measurements of portal venous blood flow (PVf), superior mesenteric artery blood flow (SMAf) and resistance (SMAR) were performed [39]. The PVf (mL/min) and SMAf (mL/min) were measured using a nonconstrictive perivascular ultrasonic transit-time flow probe (lRB, 1-mm diameter; Transonic Systems, Ithaca, NY, USA). SMAR (mmHg/mL/min/100 g body) was calculated as (MAP-PP)/SMAf.

### 4.4. Measurement of Motor Activities

Motor activities of BDL rats were determined with the Auto-Track Opto-Varimex activity monitoring system (Columbus Instruments, Columbus, OH, USA). This system is a position tracking system to detect motor activities of small laboratory rodents [40]. The traveled distance, resting time, ambulatory time and stereotypic time were separately recorded to reflect the motor activities of rats, by which reductions of motor activities indicated the aggravation of hepatic encephalopathy.

### 4.5. Hepatic Histopathological Examination, Free Cholesterol Accumulation and Fibrosis Determination

Liver tissues were fixed in 10% formalin, embedded in paraffin, sectioned in 5 μm, and stained with hematoxylin–eosin (H&E). Sirius red staining for determination of severity of liver fibrosis and filipin stain for determination of intrahepatic free cholesterol accumulation were also performed [41]. The semi-quantitative counting of filipin-positive staining droplets were measured. The H&E and Sirius red staining were examined using light microscope, and the filipin staining was observed with a fluorescence microscopy (Eclipse Ni-E, Nikon, Tokyo, Japan).

### 4.6. Intrahepatic Angiogenesis Evaluation with CD31 Immunohistochemical Staining

Liver tissues were fixed in 10% formalin and then embedded in paraffin. The liver paraffin sections were incubated with monoclonal anti-CD31 antibody (1:100, Serotec, Raleigh, NC, USA) for 1 h, which was followed by avidin–biotin complex treatment. Sections were counterstained with Mayer’s hematoxylin. Intrahepatic angiogenesis was assessed by counting the number of CD31-positive staining cells using light microscopy [39].

### 4.7. Determination of Portal-Systemic Collateral Shunting Degree

The portal-systemic shunting degree was determined using the technique described by Chojkier and Groszmann [42] and substituting color for radioactive microspheres as described previously [43].

### 4.8. Determination of the Mesenteric Vascular Density

Mesenteric angiogenesis was quantified by CD31-labeled microvascular networks in rat mesenteric connective tissue windows using immunofluorescent staining and counted by fluorescent microscopy (Eclipse Ni-E, Nikon, Tokyo, Japan) as described previously [43].

### 4.9. Determination of Systemic Lipid Peroxidation and Antioxidant Capacity

The lipid peroxidation marker of thiobarbituric acid reactive substances (TBARS) and anti-oxidant markers of glutathione peroxidase activities in the blood were measured [44]. The TBARS values were calculated with the extinction coefficients of malondialdehyde (MDA) to be 1.56 × 105 M^−1^ cm^−1^ and then expressed as nmol MDA eq. per mg protein. The TBARS activities were measure by a commercial kit (Avantor Performance Materials Taiwan Co. Ltd., HsinChu County, Taiwan). The glutathione peroxidase activities were measured using the RANSEL assay kit (Randox Laboratories Ltd., Antrim, UK).

### 4.10. Western Blot Analysis

Liver tissues were frozen in liquid nitrogen and stored at −80 ℃ until required for Western blot analysis. Blots were incubated with the primary antibody (endothelial nitric oxide synthase (eNOS) (Cell Signaling Technology, Danvers, Massachusetts, USA. 32027S; 1:1000), inducible nitric oxide synthase (iNOS) (GeneTex International Corporation, Hsinchu, Taiwan, Gtx130246; 1:1000), LOX-1 (GeneTex GTX 59636; 1:3000), NFκB (Cell Signaling 8242S; 1:3000), IκBα (Cell Signaling 4814S; 1:3000), beta-actin (Genetex Gtx629630; 1:5000)); then, the blots were incubated for 90 min with secondary antibody (horseradish peroxidase-conjugated goat anti-mouse IgG antibody; Sigma Chemical Co., St. Louis, MO, USA). The specific proteins were detected by enhanced chemiluminescence (Immobilon Western Chemiluminescent HRP Substrate, Merk Millipore Co., Billerica, MA, USA) and scanned with a computer-assisted video densitometer and digitalized system (BioSpectrum^®^ 600 Imaging System, Ultra-Violet Products Ltd., Upland, CA, USA). Then, the signal intensity (integral volume) of the appropriate band was analyzed.

### 4.11. Drugs

Alirocumab was purchased from Sanofi-Aventis Deutschland GmbH, Germany. All solutions were freshly prepared on the days of experiments.

### 4.12. Statistical Analysis

All results are expressed as mean ± standard deviation. Statistical analyses were performed using an unpaired Student’s *t*-test or ANOVA with the Least Significant Difference test as appropriate. Log-rank test was used for the survival curve analysis. Results were considered statistically significant at a two-tailed *p* value less than 0.05.

## Figures and Tables

**Table 1:** Hemodynamic parameters of sham-operated or BDL rats with or without alirocumab treatment.

	Sham + Alirocumab (*n* = 12)	Sham + Vehicle (*n* = 8)	BDL + Alirocumab (*n* = 12)	BDL + Vehicle (*n* = 11)
BW (g)	429 ± 26	420 ± 38	374 ± 23 *	384 ± 36 *
MAP (mmHg)	155 ± 9	145 ± 10	134 ± 14 *	132 ± 14 *
PP (mmHg)	8.6 ± 1.0	8.7 ± 0.8	16.4 ± 1.9 *	16.9 ± 2.6 *
HR (beats/min)	404 ± 37	394 ± 48	390 ± 34	401 ± 30
PVf (mL/min/100 g)	8.9 ± 1.3	10.1 ± 0.9	12.9 ± 2.1 *	11.7 ± 1.7 *
SMAf (mL/min/100 g)	6.2 ± 1.0	5.8 ± 0.6	8.4 ± 1.1 *	8.2 ± 1.6 *
SMAR (mmHg/mL/min/100 g)	24.2 ± 3.7	23.8 ± 2.8	14.3 ± 2.9 *	14.7 ± 4.1 *
SVR (mmHg/mL/min/100 g)	4.8 ± 0.7	5.1 ± 0.7	3.2 ± 0.5 *	3.1 ± 0.7 *
CI (mL/min/100 g)	33.4 ± 5.0	28.7 ± 2.9	43.0 ± 6.4 *	44.1 ± 7.4 *

BW: body weight; MAP: mean arterial pressure; PP: portal pressure; HR: heart rate; PVf: portal venous flow; SMAf: superior mesentery arterial flow; SMAR: superior mesentery arterial resistance; SVR: systemic vascular resistance; CI: cardiac index; Sham + alirocumab, Sham + vehicle vs. BDL + alirocumab, BDL + vehicle, * *p* < 0.05; BDL + alirocumab vs. BDL + vehicle, *p* > 0.05.

**Table 2:** Biochemistry parameters of sham-operated or BDL rats with or without alirocumab treatment.

	Sham + Alirocumab (*n* = 12)	Sham + Vehicle (*n* = 8)	BDL + Alirocumab (*n* = 12)	BDL + Vehicle (*n* = 11)
TC (mg/dL)	35 ± 11 ^a^	52 ± 12	62 ± 14 ^b^	81 ± 9 ^c^
LDL (mg/dL)	7 ± 4 ^a^	11 ± 2	31 ± 9 ^b^	45 ± 9 ^c^
Ox-LDL (ng/mL)	39 ± 5	44 ± 10	757 ± 183	714 ± 237 ^c^
Ammonia (μmol/L)	97 ± 11	106 ± 26	249 ± 86	235 ± 66 ^c^
ALT (IU/L)	45 ± 10	55 ± 12	115 ± 28	118 ± 37 ^c^
AST (IU/L)	110 ± 47	101 ± 51	620 ± 174	567 ± 132 ^c^
TB (mg/dL)	0.03 ± 0.01	0.04 ± 0.03	8.3 ± 1.1	8.4 ± 1.3 ^c^
Cr (mg/dL)	0.36 ± 0.09	0.41 ± 0.08	0.44 ± 0.08	0.44 ± 0.11

TC: total cholesterol; LDL: low-density lipoprotein; Ox-LDL: oxidized LDL; ALT: alanine aminotransferase; AST: aspartate aminotransferase; TB: total bilirubin; Cr: creatinine; ^a^: Sham + alirocumab vs. Sham + vehicle, *p* < 0.05; ^b^: BDL + alirocumab vs. BDL + vehicle, *p* < 0.05; ^c^: BDL + vehicle vs. Sham + vehicle, *p* < 0.05.

**Table 3:** Motor activities in BDL rats with or without alirocumab treatments.

	BDL + Vehicle (*n* = 11)	BDL + Alirocumab (*n* = 12)
Distance traveled (cm)	14,608 ± 4497	16,991 ± 5963
Resting time (s)	897 ± 230	802 ± 324
Ambulatory time (s)	758 ± 185	853 ± 299
Stereotypic time (s)	127 ± 47	123 ± 40

BDL + vehicle vs. BDL + alirocumab, all *p* > 0.05.
